# Cryptic Biological Invasions: a General Model of Hybridization

**DOI:** 10.1038/s41598-018-20543-6

**Published:** 2018-02-05

**Authors:** Claudio S. Quilodrán, Frédéric Austerlitz, Mathias Currat, Juan I. Montoya-Burgos

**Affiliations:** 1 Department of Zoology, University of Oxford, Oxford, OX1 3PS United Kingdom; 2 Laboratory of vertebrate evolution, Department of Genetics and Evolution, University of Geneva, Geneva, Switzerland; 3 Laboratory of anthropology, genetics and peopling history, Department of Genetics and Evolution-Anthropology Unit, University of Geneva, Geneva, Switzerland; 4 Laboratory of Eco-Anthropology and Ethnobiology, National Museum of Natural History, National Centre for Scientific Research, University Paris-Diderot, Paris, France; 5 Institute of Genetics and Genomics in Geneva (IGE3), Geneva, Switzerland

## Abstract

The dispersal of non-native genes due to hybridization is a form of cryptic invasion with growing concern in evolution and conservation. This includes the spread of transgenic genes and antibiotic resistance. To investigate how genes and phenotypes are transmitted, we developed a general model that, for the first time, considers concurrently: multiple loci, quantitative and qualitative gene expression, assortative mating, dominance/recessivity inheritance and density-dependent demographic effects. Selection acting on alleles or genotypes can also be incorporated. Our results reveal that the conclusions about how hybridization threatens a species can be biased if they are based on single-gene models, while considering two or more genes can correct this bias. We also show that demography can amplify or balance the genetic effects, evidencing the need of jointly incorporating both processes. By implementing our model in a real case, we show that mallard ducks introduced in New Zealand benefit from hybridization to replace native grey-ducks. Total displacement can take a few generations and occurs by interspecific competition and by competition between hybrids and natives, demonstrating how hybridization may facilitate biological invasions. We argue that our general model represents a powerful tool for the study of a wide range of biological and societal questions.

## Introduction

Novel breeding opportunities arise when invasive species colonize new areas and interact with related local species. The breakdown of physical reproductive barriers and subsequent hybridization has been considered either as an evolutionary process leading to the emergence of new biodiversity^1,2^ or as a threat for local taxa when induced by human activities^3^.

Hybridization represents the interbreeding between members of genetically distinguishable groups (from different populations, subspecies, species or genera)^4^. Among the possible interactions between species, hybridization has been referred to as a silent or hidden phenomenon^5,6^, with more cryptic effects as compared to other ecological interactions such as predation, competition or parasitism. Although the causes of hybridization have increased in recent years due to the translocation of invasive species^7^, the modification of natural habitats^8^, climate change^4^ and the release of genetically modified organisms^9^, the long-term consequences of hybridization on the genetics and demography of organisms are poorly understood^10^. To address this issue, we have recently defined three main types of hybridization based on the reproductive status of F_1_ offspring and the evolutionary distance between interacting taxa^11,12^. The first two types occur when distantly related species hybridize and are therefore referred to as “distant hybridization”. The first type is defined by the resulting unviable or infertile hybrids while the second type is characterized by the production of fertile hybrids but which do not undergo chromosomal recombination during meiosis. The latter type can lead to a variety of possible outcomes, such as polyploid, gynogenetic, parthenogenetic or hybridogenetic forms. The third type occurs when species are sufficiently related, so that recombination between homologous chromosomes proceeds in hybrid meiosis, resulting in at least some viable and fertile hybrids that mediate genetic introgression from one parental species to the other (referred here to as “genomic mixing”).

Hybridization involving genomic mixing induces the formation of new genetic architectures. These hybrid organisms may have lower, equivalent or higher fitness than parental individuals^8^. Hybridization occurs naturally among many plant and animal taxa and is considered an important source of evolutionary change^13^. The exact number of species that hybridize is unknown, but approximately 25% of plants and 10% of animals are known to hybridize with another related species^2^. Despite this evolutionary role in generating new species, hybridization may also represent an extinction risk with conservation concerns when it is motivated by anthropogenic factors. For instance, the introgression of non-native genes may cause the extinction of native genotypes conferring key local adaptations while introducing maladaptive alleles^14,15^. The waste of reproduction effort may reduce the likelihood of population recovery when a taxon is already threatened, which has already limited effective population size^16^. Introgression of non-indigenous genes may also change the behaviour of introgressed native individuals with unpredictable ecological effects^17^. Furthermore, the legal status of a threatened and protected species may be different for its hybrids^7,18^. However, the effects of human-induced hybridization on biodiversity are not necessarily negative, like driving species loss, as they may also increase genetic diversity and enhance the potential of adaptation to changing environmental conditions^19^.

We previously developed a model to study the two types of distant hybridization, which do not involve genomic mixing between the parental species. We demonstrated that distant hybridization represents a demographic risk for native species via wasted reproductive effort, either enhancing the effect of additional threats^12^, or directly replacing parental species by hybrid offspring^11^. Here we extended this model in order to incorporate all three types of hybridization, with a specific focus on the third type, which is characterized by the process of genomic mixing.

The general model presented here can thus be applied to any type of hybridization and to address a large range of biological questions. This approach combines important features of population genetics and population ecology to explore the potential outputs of a hybrid system. The interactions between genetic and demographic factors have rarely been incorporated together in earlier modelling efforts^20^. Previous attempts at modelling hybridization with genomic mixing have focused on quantitative gene expression, ignoring the influence of density-dependent demographic effects^21–23^. The few approaches that have considered density-dependent effects simulated a single locus^24–26^. We present here the first general model fully addressing six important processes that affect the impact of hybridization on biodiversity: (1) intra- and inter-specific density-dependent competition; (2) the degree of dominance/recessivity of alleles in hybrids; (3) assortative mating between interacting organisms; (4) the involvement of multiple loci; (5) quantitative and qualitative gene expressions; and (6) the possibility to simulate both neutral and selective mechanisms.

By using our model, we addressed two main questions about hybridization and cryptic genetic invasions: (i) how do interactions between genetics and demography influence the observed phenotypic landscape when hybridization occurs? (ii) how can native organisms be threatened by hybridization with an invasive species? We finally illustrate the usefulness of our model by analysing real situations in which the hybridization of mallard ducks (*Anas platyrhynchos*) is a conservation concern.

## Results

### Effects of the number of genes considered

**Figure 1:**
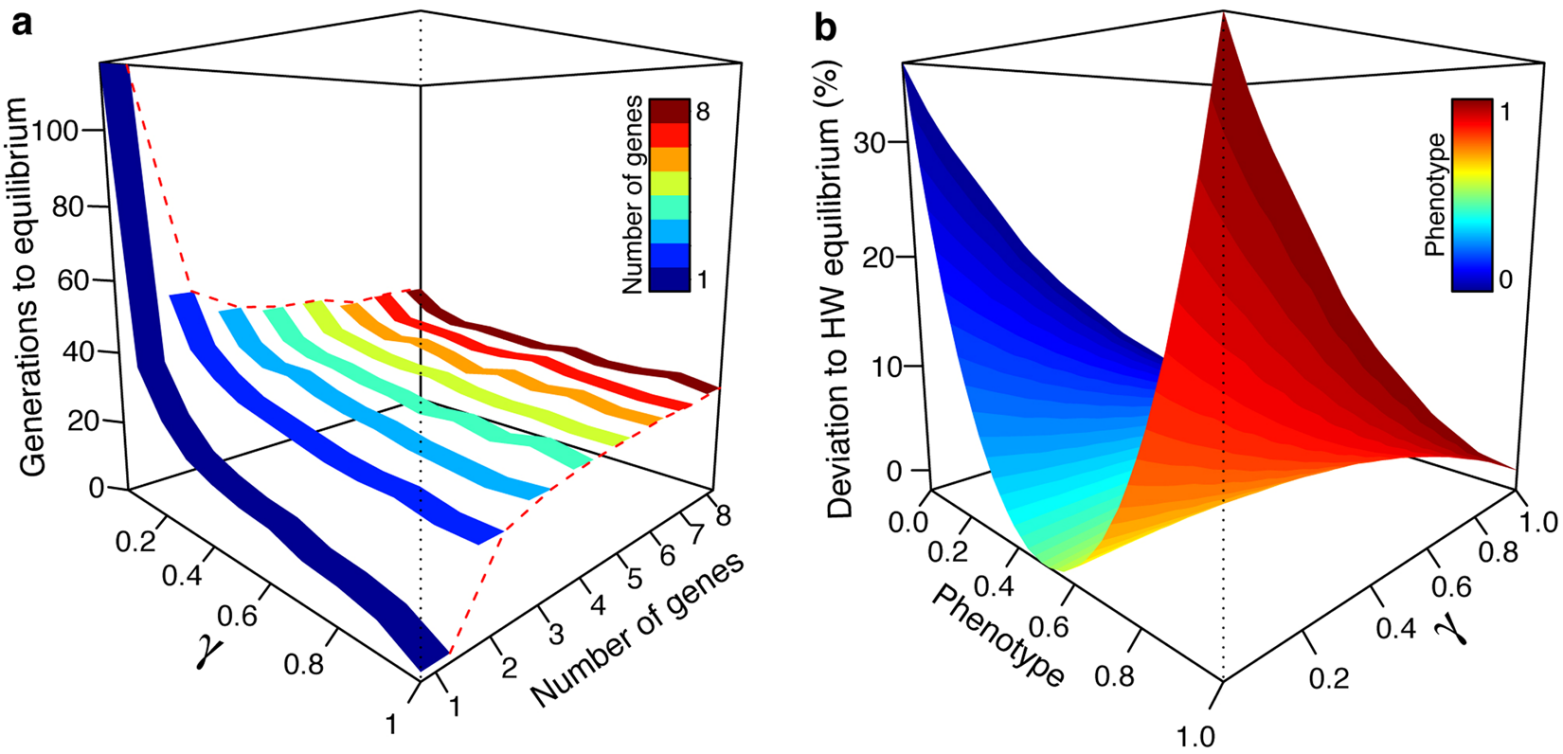
Simulation results when multiple genes are considered in the model. (**a**) Effect of the number of genes and interbreeding success rate (*γ*) on the time to reach frequencies in equilibrium; (**b**) Influence of interbreeding success rate (*γ*) on the deviation from Hardy-Weinberg (HW) equilibrium of five biallelic genes (simulations with one, two or three genes are available in Supplementary Fig. S1). We explored various levels of interbreeding success rate, from rare hybridization success (*γ* = 0.01) to a panmictic mating system (*γ* = 1). The data presented was obtained after 150 generations of genomic mixing.

We first assessed the influence of the number of simulated genes on the time to reach frequencies in equilibrium and measure the deviation from Hardy-Weinberg (HW) equilibrium at this time (Fig. 1). We performed these simulations without incorporating the density-dependent demographic effects in order to get relative frequencies, illustrating the performance of the model independently of specific demographic values (see equation 13, Methods).

We found that the time at which equilibrium is reached depends on the level of interbreeding success rate (*γ*) and also on the number of genes considered. Simulations with a single locus are extremely influenced by *γ*. When comparing single locus simulations with those having more than three genes, a small value of *γ* (0.01) tends to increase the number of generations to reach the equilibrium by ~1.8 times while panmictic reproduction (*γ* = 1) tends to decrease the number of generations needed by >10 times. The number of generations needed to reach frequencies in equilibrium is stable when more than two genes are considered in the simulations, especially with *γ* larger than 0.2 (Fig. 1a). There is still approximately 0.3 to 0.4 times of difference to reach equilibrium when using two genes compared to three genes.

The deviation from HW equilibrium at the time of frequencies in equilibrium is also affected by the interbreeding success rate (Fig. 1b). We illustrate this result using a quantitative character depending on five biallelic genes (from red to blue, Fig. 1b). A maximum deviation of approximately 40% is reached with a small interbreeding success rate (*γ* = 0.01) if the phenotype values are extreme (0.0 or 1.0), with an asymptotic decrease with increasing values of interbreeding. Similar results are found with two simulated genes or more (Supplementary Fig. S1). However, the deviation is much higher with a single biallelic gene (Supplementary Fig. S1).

These results highlight the importance of considering multiple genes in the simulations when a given trait is suspected to be controlled by multiple genes. Because the number of generations to reach frequencies in equilibrium, and the deviation from HW equilibrium, are very stable when simulating three or more genes (Fig. 1a and Supplementary Fig. S1), we conclude that a set of three genes gives a good approximation to the results obtained with more than three genes. Thus, we recommend the use of three genes when the number of additive neutral genes controlling a quantitative trait is unknown. Simulating more genes will give very similar results but will take more computational time.

### Exploration of the phenotypic landscape (additive trait)

The phenotypic landscape of an additive trait is extrapolated using five biallelic genes. We incorporate the density-dependent effects in this analysis using parameters drawn from the population dynamics of mallards (see equation 13). In our simulations, we let the two parental taxa *N*_0_ and *N*_1_ evolve independently during the first 100 generations, then demographic and genetic interactions among the two taxa were simulated for an additional 150 generations. Depending on the case, the parental taxa may or may not be in competition for environmental resources. However, in all cases, we let competition occur among individuals within the same class and also among classes, according to the fraction of shared parental loci.

**Figure 2:**
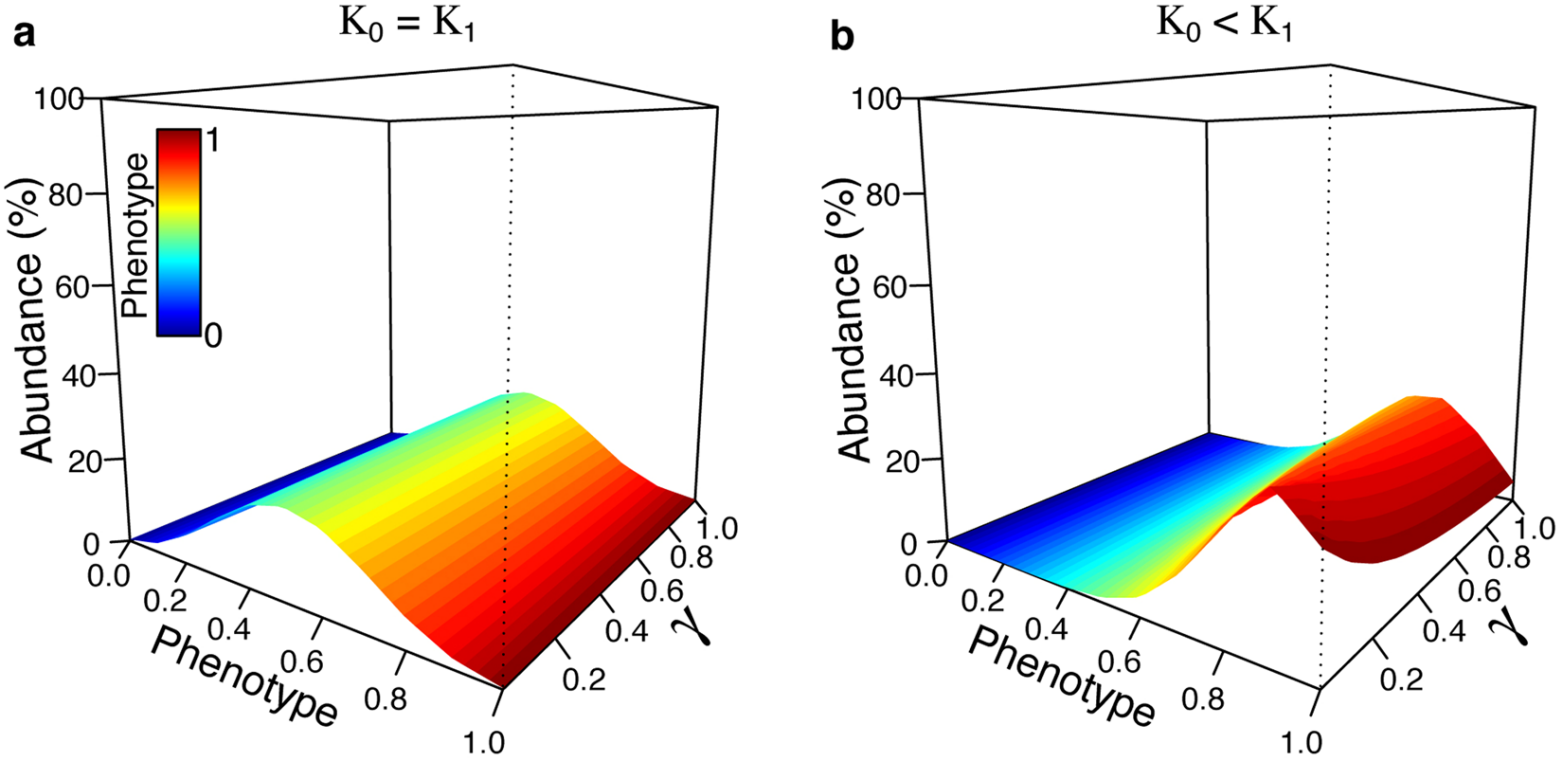
Density-dependent demographic parameters influencing the landscape of observed phenotypes. The phenotypic landscape is extrapolated from the contribution of five additive genes (*n*_*L*_ = 5). We show the effect of the absence of interspecific competition (α_01_ = α_10_ = 0) with equal or unequal carrying capacities between the two parental species (*K*_1_ = *K*_0_ or *K*_1_ = 1.5 *K*_0_). We explored various levels of interbreeding success rate, from rare hybridization success (*γ* = 0.01) to a panmictic mating system (*γ* = 1). The data corresponds to the situation after 100 generations of independent evolution and 150 generations of hybridization (250 total generations).

With identical demographic parameters, the expected phenotypic landscape depicts a Gaussian-like shape with higher abundance in the most heterologous class, increasing genetic diversity (0.5, Fig. 2a). This pattern is perturbed by asymmetrical carrying capacities between parental taxa and by competition. The phenotype with highest abundance shifts towards parental class 1 when adding 50% more carrying capacity to this class (*K*_1_ = 1.5 *K*_0_, Fig. 2b). The optimum is still present in individuals with hybrid genomes, but in those with more genes shared with parental class 1. Even though there is no competition between parental taxa, class 0 and hybrids with more alleles shared with it, are almost extinct due to the competitive exclusion of hybrids with more alleles shared with taxon 1. When adding competition between parental taxa, parental class 0 and all hybrid categories becomes extinct and the community is thus composed exclusively of individuals of class 1 (Supplementary Fig. S2). Overall, these results show that hybridization may exclude a native species having a demographic disadvantage compared to the other taxon. This effect is indirect through the hybrids in absence of competition between parental taxa and stronger if there is competition between them, in both cases facilitating the invasion of non-native genes.

### Adding Selection

**Figure 3:**
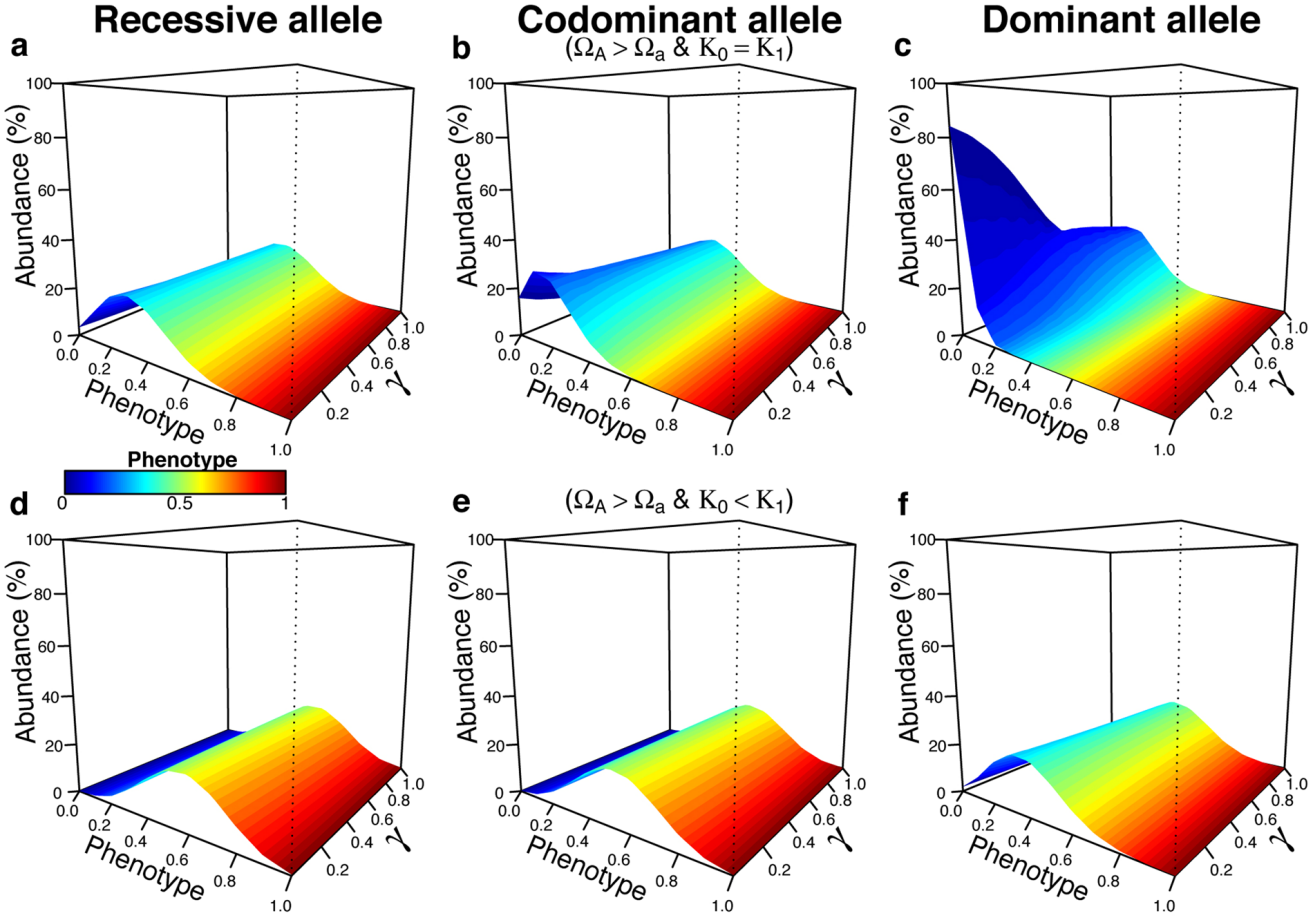
Effects of a deleterious allele “*a*” with fitness reduced by 30%. The phenotypic landscape is extrapolated from five additive genes (*n*_*L*_ = 5). There is no interspecific competition (α_01_ = α_10_ = 0) and carrying capacity may either be equal or unequal between the interacting taxa (*K*_1_ = *K*_0_ or *K*_1_ = 1.5 *K*_0_). (**a**) Allele “*a*” is recessive (*ε*_*a*_ = 0); (**b**) allele “*a*” is co-dominant (*ε*_*a*_ = 0.5); (**c**) allele “*a*” is dominant (*ε*_*a*_ = 1). Simulations incorporating interspecific competition (α_01_ = α_10_ ≠ 0) are available in Supplementary Fig. S3. We explored various levels of interbreeding success rate, from rare hybridization success (*γ* = 0.01) to a panmictic mating system (*γ* = 1). The data corresponds to the situation after 100 generations of independent evolution and 150 generations of genomic mixing (250 total generations).

We incorporated the effect of purifying selection by decreasing the fitness (*ω*) of individuals carrying a specific co-dominant allele “*a*”, originally present in parental class 1. This may represent the effect of higher susceptibility to a disease or any other negative selective pressure. We first assumed that parental classes are not in competition for environmental resources (α_01_ = α_10_ = 0). When carrying capacity is equal between both parental taxa and the allele “*a*” is recessive (*ε*_*a*_ = 0), the maximum abundances are found in the hybrid classes close to class 0. Increasing the interbreeding success rate shifts this optimum approximately 10% away from class 0, towards more heterologous classes (from 0.3 to 0.4) (Fig. 3a). This effect is stronger with co-dominant alleles (*ε*_*a*_ = 0.5). The classes with the highest frequency are closer to parental class 0 than previously (*i*~0.1). The interbreeding success rate moves this optimum to about 20% (to *i*~0.3) when the reproduction is panmictic (γ = 1) compared to a low interbreeding rate (γ = 0.01) (Fig. 3b). When this allele is dominant (*ε*_*a*_ = 1) and the interbreeding success rate is small (*γ* = 0.01), the maximum abundance is found in parental class 0 (~80%), but increasing values of interbreeding success rate shifts this optimum away of class 0 of about 10%, to heterologous hybrid classes *i~*0.1 (Fig. 3c). Whatever the scenarios of dominance/recessivity inheritance for allele “*a*”, individuals of class 1, initially carrying this allele, and those with a close genotype become extinct. A deleterious allele bared by one species thus brings this species to the verge of extinction despite admixture with another species that does not carry this deleterious allele.

The effect of purifying selection in allele “*a*” may be counterbalanced by a demographic advantage given to the species originally carrying this allele (*K*_1_ = 1.5 x *K*_0_). The number of hybrid classes with more genes shared with 1 slightly increases with a larger interbreeding success rate, for all scenarios of dominance/recessivity inheritance, even if parental class 1 is still rare in the whole community (Fig. 3d–f).

Using the same settings as before (with species 1 having a higher carrying capacity and being affected by purifying selection in allele “*a*”), we added competition between parental taxa (Supplementary Fig. S3). Hybrid categories with the highest proportion of genes shared with class 1 are favoured when allele “*a*” is recessive or when it is co-dominant with high values of interbreeding success rate (*γ* > 0.3). There is still a complete exclusion of individuals of class 1 when allele “*a*” is dominant and interbreeding success rate is smaller than 90% (*γ* < 0.9). Otherwise only class 0 remains in the community. Completely opposite results can be obtained when increasing further the carrying capacity of class 1 (*K*_1_ = 2*K*_0_). Individuals of this parental class are able to completely exclude all other categories, independently of the dominance/recessivity inheritance of the deleterious allele “*a*” (Supplementary Fig. S3).

**Figure 4:**
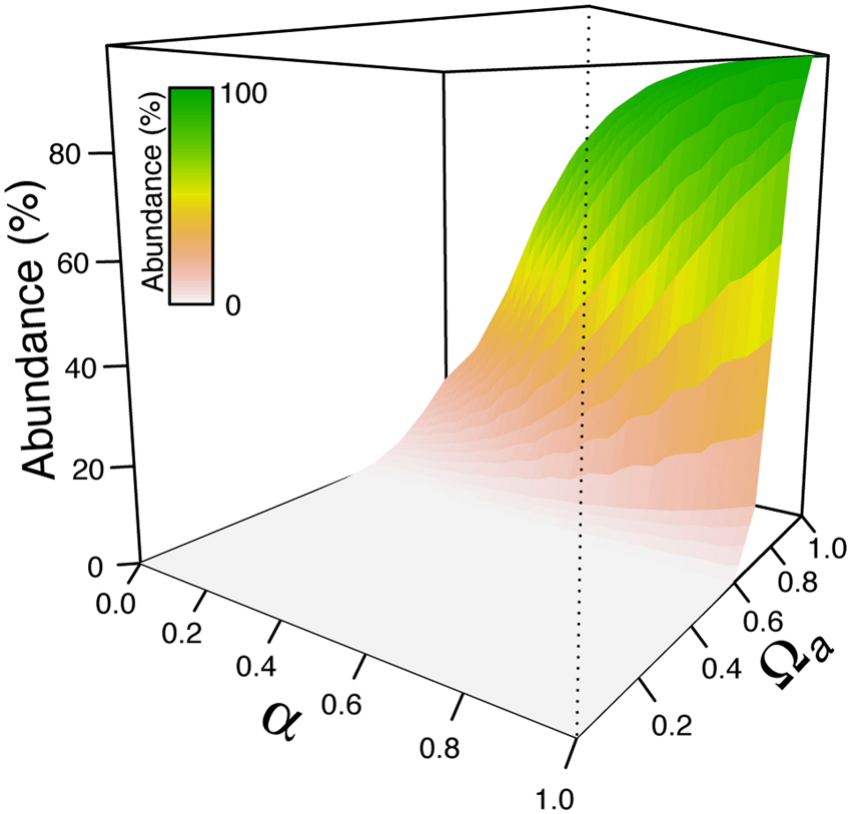
Interspecific competition and purifying selection. We show the combined effect of interspecific competition (α_01_ = α_10_ = α) and purifying selection on the abundance of a parental species of class “1” with higher carrying capacity (*K*_1_ = 2 *K*_0_), but affected by a reduced fitness of allele “*a*” (*n*_*L*_ = 5, data obtained after 100 generations of independent evolution and 150 generations of hybridization).

We illustrate the relation between interspecific competition (*α*) and reduced allelic fitness in Fig. 4. In this simulation, the parental species of class 1 has twice the carrying capacity than the species of class 0 (*K*_1_ = 2 *K*_0_), but is affected by various levels of fitness reduction in the allele “*a*”. Individuals of class 1 are the most abundant in the community if the reduction in fitness is smaller than 20% (Ω_A_ > 0.8) and the competition between parental taxa is higher than 70% (*α* > 0.7). The competitive advantage of having higher carrying capacity does not compensate for a reduction in fitness over 40% (Ω_A_ < 0.6), in which case species of class 1 become extremely rare in the community (Fig. 4). These results stress that both genetics and demography are important to shape the observed phenotypic landscape and its optimum and that one may either increase or cancel the effects of the other.

The landscape of possible phenotypes is unimodal in the previous simulations, but multimodal optimums are also possible. To illustrate this, we used our model to recognize a specific genotype within a phenotypic class (Supplementary Fig. S4). We additionally simulate two taxa in competition and differentiated by three biallelic genes. Both species were allopatric during the first 100 generations. The phenotypic landscape turns into a Gaussian-like distribution after a few generations of panmictic interbreeding (*γ* = 1). In generation 200, the genotype “*AaBbCc*” starts to be lethal in our simulations and the phenotypic landscape turns into a bimodal shape. Other multimodal forms can be obtained by adding various lethal genotypes or more than a single allele with reduced fitness. This whole exploration phase demonstrates how useful can be our model to study the outcomes of many potential complex situations of hybridization involving many evolutionary factors.

### Fitting observed values of hybridization between mallards and grey ducks

We applied our model to the colonization of mallards in New Zealand, where they hybridize with native grey ducks. This last species was abundant at the beginning of the 20^th^ Century but is seriously threatened nowadays. The decrease of this native species and the appearance of hybrid phenotypes are well documented. There are seven phenotypes previously defined by a morphology that we assume are the expression of three additive genes (2*n*_*L*_ + 1) and for which the relative frequencies are documented. The aggressive competition and the use of generalist habitats have been documented in invasive mallards. In order to explain the observed hybrid frequencies, we thus set full interspecific competition of mallards (α_mallard_ = 1) to assess the carrying capacity (*K*) and the amount of interspecific competition performed by the native species (α_native_).

**Figure 5:**
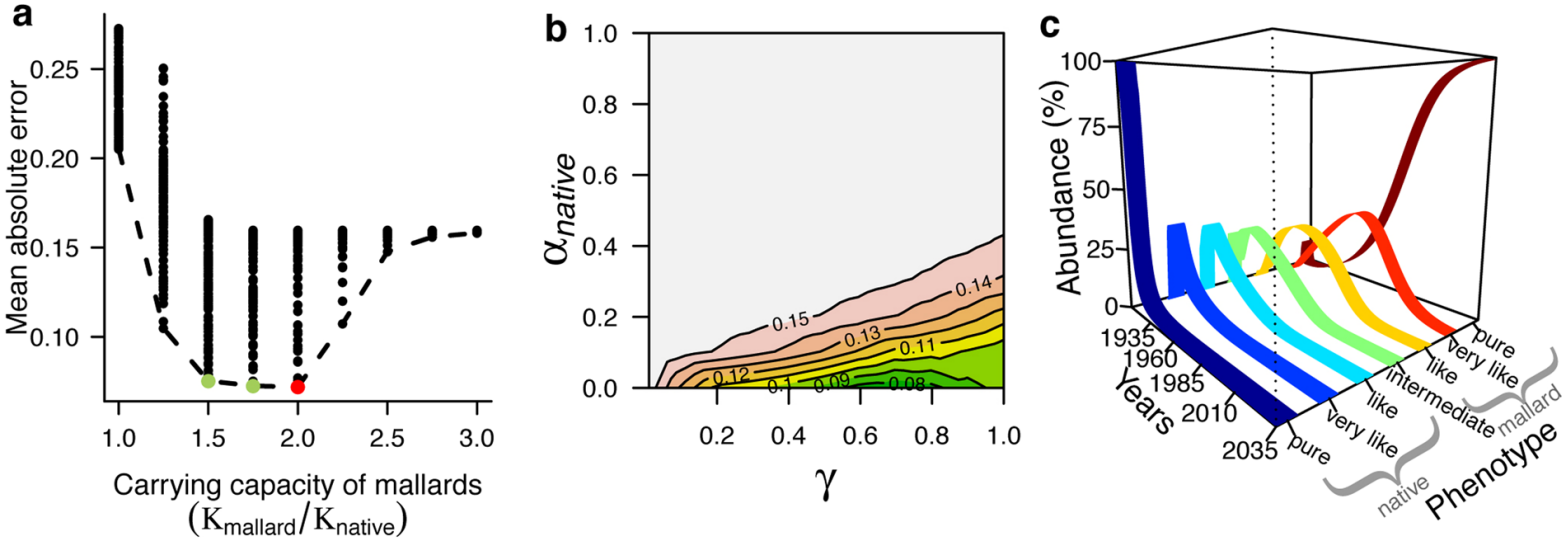
Demographic declines of a native species. Simulations are based on the colonization history of mallards (*Anas platyrhynchos*) in New Zealand where they seriously threaten the native grey duck (*Anas superciliosa*). We evaluated different values of carrying capacity (*K*), interspecific competition exerted by the native species (α_native_) and interbreeding success rate (*γ*) to minimize the error when fitting observed abundance values. There are seven documented phenotypes due to hybridization between mallards and grey ducks. We assume these phenotypes are the product of three additive genes (*n*_*L*_ = 3, see Methods). (**a**) Relative carrying capacities of mallards to explain the colonization time in New Zealand (red point minimize the error with observed values); (**b**) mean absolute error when fixing the best estimated carrying capacity of mallards (two times larger) and varying the value of competition (α_native_) and interbreeding success rate (*γ*) (the dark green values minimize the error: α_native_ = 0 and *γ* = 0.7); we explored various levels of interbreeding success rate, from rare hybridization success (*γ* = 0.01) to a complete panmictic mating system (*γ* = 1); (**c**) forward simulation using the best combination of genetic and demographic parameters that explain the invasion of mallards in New Zealand. Simulations with alternative carrying capacities (green points in Fig. 5a) are presented in Supplementary Fig. S5.

We first estimated the carrying capacity that best explains the invasion of mallards to be between 1.5 to twice that of the grey duck species, leaving all other parameters free to vary (Fig. 5a). We then set the carrying capacity of mallards to be twice as large as that of grey ducks for the subsequent simulations, because it presents the minimum mean absolute error in explaining the observed hybrid frequencies (<7%). Second, we estimated that high values of interbreeding success rate (*γ* > 0.4) and high asymmetrical competition between native and invasive species (α_native_ ≪ α_mallard_) are necessary to explain the invasion of mallards in New Zealand. The best combination of parameters supports an absence of competition from grey duck to mallard (α_native_ = 0) and 70% of interbreeding success rate between both taxa (Fig. 5b).

We used these estimated parameter values to explore the phenotypic evolution due to the invasion of mallards since 1910, when a stable feral population was first observed (Fig. 5c). The native species is dominant at the beginning of the simulations, but hybrids with a proportion of shared genes closer to the native species become quite abundant (~25%) in a few generations. They were in competition with the native species provoking an asymptotical population decrease through time. These hybrids also started to disappear in favour of other hybrid classes with a proportion of shared genes closer to mallards. An explosion in the abundance of mallards occurred when the native duck was already rare, due to competition with hybrids.

Similar results are obtained when performing these simulations with the best combination of parameters for carrying capacities 1.5 and 1.75 times larger for mallards (Supplementary Fig. S5). The hybrids with genotypes closer to the native species are the first that start to be abundant, but also the first to decline after the colonization of mallards. The final projections 20 years from now always result in the exclusion of the native species and hybrids by mallards. These simulations illustrate the potential of hybridization as a process that facilitates biological invasions.

## Discussion

### Development of a general model of hybridization

We developed a general model of hybridization that can be applied to a large range of ecological and evolutionary case studies. Here the main focus of the model is placed on genomic mixing and the appearance of new hybrid phenotypes, but it could also allow the study of outbreeding depression due to the appearance of infertile hybrids or the production of polyploidy and hybridogenetic forms^11,12^.

Our general model was developed by using our previously published distant species hybridization model to which we incorporated a modified version of the approximate hypergeometric model, originally developed to study intraspecific quantitative genetic characters^27,28^. Our approach allows for the study of interspecific genetic interactions and the recognition of specific alleles or genotypes within genetic classes, leading to the incorporation of both quantitative and qualitative gene expression. Therefore, further applications of our model are not limited to quantitative phenotypes, and can be extended to the proportion of parental genomes, assessing the effects of additive genes and the influence of specific loci. In this last case, models involving only genotypes can be highly computationally demanding due to the number of genotypic classes increasing exponentially with the number of loci (2^nL^). Our approach thus simplifies the simulation of any number of genes by hugely reducing the number of possible classes (2n_L_ + 1) and by recognizing the alleles or genotypes of specific interest.

Other applications of the approximate hypergeometric model have assessed the effect of hybridization between genetically distinct groups on quantitative traits^21,23^. However, our model is the first to incorporate density-dependent demographic effects, recognize target genotypes or alleles and incorporate dominance/recessivity inheritance. These are major improvements that allowed us to examine the effect of selection in our analysis. We illustrated the effect of deleterious alleles, but the influence of positive and balancing selection may also be explored in future studies.

### Incorporation of multiple genes in the simulations

Despite the main influence of population dynamic on the outcomes of hybridization with introgression, only a few previous approaches have incorporated density-dependent effects, mainly taking into consideration a single biallelic gene^24,26^. Simulations with a single gene may reveal general patterns^25^, but we showed that they may significantly bias conclusions about the ecological and evolutionary consequences of hybridization. By simulating multiple genes, we demonstrated that the time to reach equilibrium frequencies and the level of deviation from Hardy-Weinberg equilibrium are affected by the number of simulated genes, obtaining more stable patterns when simulating more than two genes. This may seriously affect the estimation of extinction risk. It is thus important to simulate multiple genes when aiming to investigate processes with an unknown number of genes involved.

### Genomic mixing and conservation

The hybridization between genetically distinct organisms may influence the appearance of new genotypic architectures. The shape of the phenotypic landscape depends on a combination of demographic factors (e.g. carrying capacities and competition) and genetics (e.g. dominance/recessivity inheritance and selection). We demonstrated that these novel phenotypes could become fixed in the community replacing one or both pure parental genotypes by a hybrid swarm or progressively disappearing, thereby facilitating the invasion of one of the parental genotypes. Moreover, we demonstrated that the influence of detrimental alleles might be counterbalanced by larger carrying capacities or higher competitive skills. A mixture of both genetic and demographic effects is thus important to explain the optimum phenotypes susceptible to be observed in the field. Further application may help to fill the gap between population ecology and population genetics, disciplines that have been recognized to complement each other but that have evolved separately^20^.

### New opportunities brought by hybridization

Determining whether hybridization cases are natural or human-induced is a high priority in conservation biology^3^. In this last case, the loss of important local adaptations due to genetic swamping, the behavioural change influencing ecological interactions, and the reduced likelihood of population recovery due to reduced reproductive value, may certainly represent a serious threat for native species^14^. However, even when hybridization is induced by humans, it is not necessarily a threat for native biodiversity. Indeed, hybridization is a source of genetic diversity that may be important for adaptation to changing environmental conditions, particularly to prevent the negative effects of habitats that are modified by humans. For instance, the novel genetic architecture visible in our phenotypic landscapes may also represent the increase of genetic diversity brought by hybridization. We exposed different situations in which theoretical parental taxa admix and generate new hybrid individuals that may be found at high frequencies. If those hybrids do not have a much-reduced fitness and do not undergo a change in ecological functions as compared to their parental organisms, hybridization may simply represent an increase of genetic diversity. Additionally, we differentiated parental phenotypes with a colour pattern (blue or red). However, the range of colours may also represent specific niches, with one potentially available to new hybrid organisms (e.g. green), in which case hybridization is driving new opportunities of adaption and evolution^29^. In our case study, the hybridization with invasive mallards is mainly considered a detrimental threat to native species, but the occurrence of novel possibilities of adaptation has already been proposed for the North American mottled duck (*Anas fulvigula*), which has increased its genetic diversity due to hybridization with mallards^30^. The negative or positive effect of hybridization on biodiversity is therefore not only determined by human actions, but also by the resulting consequence on fitness and by the alteration of key ecological interactions. Assisted hybridization may eventually represent a conservation management tool to facilitate local adaption to climate change^4,19^ or to rescue small populations affected by inbreeding depression^14^. Our model could thus be an ideal tool in the future to assess the effects of hybridization on biodiversity.

### Consequences of mallard invasion in New Zealand

We showed that the appearance of hybrids might threaten native taxa and even facilitate a biological invasion. Our results suggest that mallards have taken advantage of hybridization to succeed a rapid expansion in novel areas. Their aggressive interspecific competition and their capacity to exploit new resources related to human-disturbed environments^31^ provide mallards with higher opportunities to displace native taxa by direct competition or indirectly through competition with hybrids. In New Zealand, there is no evidence of decreased hybrid fertility between grey ducks and mallards^32^. Our simulations estimate a competitive pressure first exerted by grey-like hybrids and then by mallard-like hybrids and mallards themselves. The final expectation is the extinction of grey ducks and hybrids in favour of mallards. Even though these simulations were based on frequencies observed during the 80’s^33^, while grey ducks are currently no more considered as game species but as an endangered one^34^, our simulations fit well with present-day observations. Grey ducks are extremely rare, with some populations being already extinct and the majority of individuals are mallard-like hybrids or pure mallards^31,32^. The current extinction risk is so high that the most efficient strategy would be to translocate the few remaining grey ducks to an offshore island without mallards^32^.

## Conclusions

Our general model opens new research opportunities in ecology, conservation and evolution because it is the first approach combining density-dependent demographic effects with quantitative and qualitative multigenic simulations. Through simulations based on our model, we highlighted the potential appearance of novel genetic architectures that may subsequently disappear or be fixed in the community. These novel genotypes may threaten native taxa or be a source of genetic diversification potentially leading to adaptation to new environmental conditions. Our case study was based on mallards, but further studies with specific parameter values may explore the effect of hybridization on any diploid organism. The *R* script of our modelling approach is freely available at: http://genev.unige.ch/montoya-currat/scripts/. It is easily modifiable to fit particular case studies with specific parameter values or to investigate other theoretical questions about hybridization. Further studies may, for instance, consider hybridization success rate as a variable instead of a fixed parameter, which may depend on demographic frequencies or given environmental conditions. More complex demographic models, incorporating environmental and demographic stochasticity, can also be envisaged in order to maximize the likelihood to explain historical datasets^35,36^. New hybridization opportunities induced by humans are expected for the near future either due to the invasion of translocated species^37^, domesticated animals^16,38^ and genetically modified organisms^9^, or due to the range expansion of species induced by climate change^4,6^. This general model of hybridization represents an important instrument for the development of conservation strategies and for the understanding of processes behind the emergence and evolution of new species.

## Methods

### General description of the model

Our model describes the genetic consequences of the interbreeding between a species (*N*_1_) and a closely related taxon (*N*_0_). One of them usually represents newly arrived organisms. The community is composed of diploid parental taxa, initially differentiated by multiple independent biallelic loci and assuming normal recombination between homologous chromosomes, with case dependent interbreeding rates.

We used an approximate hypergeometric model to decompose the possible genotype space in a discrete number of genetic classes, representing the proportions of loci coming from each parental species. This model was originally developed to study additive genes in a discrete number of phenotypes^27,28^, but the approach presented here allows the investigation of specific alleles or genotypes within groups. It is therefore not limited to quantitative characters but extends the method to the analysis of qualitative traits.

The number of independent biallelic loci that determine the reproductive isolation between both species is *n*_*L*_. In each locus, alleles are denoted by upper case (*AA, BB, CC…)* if it comes from *N*_0_ and by lower case (*aa, bb, cc…*) if it comes from *N*_1_. We assume a genotypic space between both taxa ranging between 0 and 1, in which 0 codifies the parental *N*_0_ genotype and 1 the parental *N*_1_ genotype. The number of possible hybrid classes is 2*n*_*L*_ − 1. Therefore, *N*_*i*_ for $\tfrac{1}{2{n}_{L}}\le i\le 1-\tfrac{1}{2{n}_{L}}$ are hybrids in which the proportion of upper or lower cases denote the proportion of genes coming from *N*_0_ or *N*_1_^21^.

The mating frequency of individuals of a given genetic class within or between classes, compared to all possible combinations, determines the contribution of each class to the next generation. Thus the probability *M*_*ij*_ for each female of class *i* to mate with a male of class *j*, for all *i*,*j* *∈* [0, …, 1] is:

1 $${M}_{ij(t)}=\frac{{{\gamma }}_{ij}{N}_{j(t)}}{{\phi }_{i(t)}}$$

Where *φ*_*i*_ is a normalization factor such that Σ _*i*_ *M*_*ij*_ = 1. In our model, the parameter *γ*_*ij*_ represents the interbreeding success rate between individuals of class *i* and *j*, as introduced by Currat, *et al*.^5^ and modified by Quilodrán, *et al*.^12^. This parameter represents any barrier to the successful reproduction between organisms *i* and *j*, as either: (i) prezygotic, in which case the value of 1 − *γ*_*ij*_ may be seen as a measure of assortative mating; (ii) postzygotic, where *γ*_*ij*_ could represent offspring viability or relative fitness; or (iii) a combination of prezygotic and postzgotic barriers. In any case, when *γ*_*ij*_ = *γ*_*ji*_, the interbreeding success rate is symmetrical between both classes, while it is asymmetrical when *γ*_*ij*_ ≠ *γ*_*ji*_. When *γ*_*ij*_ = *γ*_*ji*_ = 0, there is no successful hybridization between *i* and *j*, whereas a value of 1 corresponds to a complete panmictic mating^39^. Any other value of *γ*_*ij*_ *∈* [0,1] implies that mating is locally nonrandom and reproduction occurs more often between members of class *i* than between individuals of different classes *i* and *j*. The interbreeding success rate could be fixed among phenotype classes (each *γ*_*ij*_ is set a priori), or variable depending on the proportion of genes coming from both parental species. In this latter case, for all *i,j* *∈* [0, …, 1], the interbreeding success rate is computed as:

2 $${\gamma }_{ij}=\sum _{u=0}^{2}\sum _{q=0}^{2}\frac{1}{2}(2-|u-q|(1-\gamma ))\frac{{f}^{u}{f}^{q}(\begin{array}{c}2{n}_{L}-2\\ 2{n}_{L}i-u\end{array})(\begin{array}{c}2{n}_{L}-2\\ 2{n}_{L}\,j-q\end{array})}{(\begin{array}{c}2{n}_{L}\\ 2{n}_{L}i\end{array})(\begin{array}{c}2{n}_{L}\\ 2{n}_{L}\,j\end{array})}$$

The first section on the left side of equation (2) represents the strength of interbreeding between and within genetic classes. Reproduction is panmictic between individuals with the same genotype, but the interbreeding success rate decreases with the loss of shared loci within and between classes. For instance, the reproduction between two individuals with 50% of shared genes from parental species, in a genotype space defined by five genes (*i* = 0.5, *j* = 0.5, *n*_*L*_ = 5), would be panmictic if both have the same genome (e.g. “*AaBbCcDdEe*”), but the amount of interbreeding decreases when the genomes are different (e.g. “*AaBbCcDdEe*” and “*AABBCcddee*”); and even more when they come from different classes (e.g. *j* = 0.2, “*AAbbccddee*”). The values of “*u*” and “*q*” represent the frequency of parental genes at a single locus. They take values of 0, 1 and 2 depending on the number of lower case alleles. The second section, on the right side of equation (2), is a combinatory factor to take into account all loci in the analysis. The value of “*f*^*u*^” (and “*f*^*q*^”) represents the expected genotype frequency under Hardy-Weinberg (HW) equilibrium for each locus (i.e. 1:2:1 for a biallelic gene if the two alleles are in equal frequencies, e.g.: aa:2Aa:AA), and it is computed as: $\tfrac{3-{(-1)}^{u}}{2}$. The frequencies of genetic classes are therefore at HW equilibrium when the reproduction between parental classes is panmictic (*γ*_01_ = *γ*_10_ = 1).

Equation (2) assumes that gene flow between parental species is symmetrical (*γ*_01_ = *γ*_10_ = *γ*). Asymmetrical gene flow between parental species (*γ*_01_≠*γ*_10_), due for instance to sex-biased mating preference or sex-biased survival of hybrids, may also be incorporated by setting *γ* = *γ*_01_(1 − *i*) + *γ*_10_*i*, with hybrids behaving according to the proportion of genes coming from parental species.

We then compute the number of breeding pairs composed of females of class *i* and males of class *j*, yielding offspring of class *k* as:

3 $${B}_{ij,k(t)}={N}_{i(t)}\,{M}_{ij(t)}{C}_{ij,k}{\omega }_{k}$$

Where *C*_*ij,k*_ is the proportion of offspring of class *k* resulting from a reproduction event between individuals of class *i* and *j* relative to all progeny produced by *i* x *j*. These probabilities were presented by Doebeli^28^, and were modified by Ferdy and Austerlitz^21^ (in their equations 2, 3 and 4) to work with proportional phenotype classes, as follows:

4 $${C}_{ij,k}=\sum _{g,h\in [0,\mathrm{...},1]}pro{b}_{i}(g)pro{b}_{j}(h)b(g,h,k)$$

*prob*_*i*_(*g*) is the probability of two individuals belonging to different classes *i* and *g* to have an intragenomic overlap, which is the proportion of alleles coming from the same parental species (i.e. the number of loci for which both alleles are lower cases); it is computed as:

5 $$pro{b}_{i}(g)=[(\begin{array}{c}2{n}_{L}\\ 2{n}_{L}g\end{array})/(\begin{array}{c}2{n}_{L}\\ 2{n}_{L}i\end{array})]\sum _{s=0}^{2{n}_{L}(i-2g)}(\begin{array}{c}{n}_{L}-2{n}_{L}\,g\\ s\end{array})(\begin{array}{c}{n}_{L}-2{n}_{L}\,g-s\\ 2{n}_{L}i-4{n}_{L}\,g-s\end{array})$$

and *b*(*g*,*h*,*k*) is the probability that crosses between *g* and *h* yield individuals of classes *k*, it is calculated as:

6 $$b(g,h,k)=(\begin{array}{c}2{n}_{L}i-4{n}_{L}\,g+2{n}_{L}\,j-4{n}_{L}h\\ 2{n}_{L}k-2{n}_{L}\,g-2{n}_{L}h\end{array}){(\frac{1}{2})}^{2{n}_{L}i-4{n}_{L}g+2{n}_{L}j-4{n}_{L}h}$$

We introduce the parameter *ω*_*k*_ (equation 3), which represents an inherited trait related to fitness in offspring of type *k*. It could represent the resistance to a particular disease, the relative survival to predation, or any other factor that confers a selective advantage, and that is genetically inherited. The value of *ω*_*k*_ is assumed to be expressed by one independent biallelic locus, in which both parental species are homozygous. Therefore, the expression of this character in the resulting phenotype class *k* depends on (1) the probability to be heterozygous (*ρ*_*Aa*_) or homozygous (*ρ*_*AA*_ or *ρ*_*aa*_) for this locus and (2) the dominance degree of the alleles “*A*” (*ε*_*A*_) or “*a*” (*ε*_*a*_). For individuals of class *k* *∈* [0, …, 1], it is computed as:

7 $${\omega }_{k}={{\rm{\Omega }}}_{A}{\rho }_{AA,k}+({\varepsilon }_{A}{{\rm{\Omega }}}_{A}+{\varepsilon }_{a}{{\rm{\Omega }}}_{a}){\rho }_{Aa,k}+{{\rm{\Omega }}}_{a}{\rho }_{aa,k}$$

With *ε*_*A*_ + *ε*_*a*_ = 1. For instance, if *ε*_*A*_ = 1 and *ε*_*a*_ = 0, a character carrying the “*A*” allele is dominant while a character carrying the “a” allele is recessive. If *ε*_*A*_ = *ε*_*a*_ = 0.5, both characters are codominant. The values of Ω_*A*_ and Ω_*a*_ are parameters that represent the relative fitness at the allelic level, in which the allele with the highest fitness has Ω = 1, while the other one is expressed as a fraction relative to 1.

The probability of being homozygous for an upper case allele “*A*” (*ρ*_*AA*_) for any individual of class *i*, with *n*_*L*_ number of genes, is calculated as:

8 $${\rho }_{AA,i}^{{n}_{L}}=(\begin{array}{c}2{n}_{L}-2\\ 2{n}_{L}i\end{array})/(\begin{array}{c}2{n}_{L}\\ 2{n}_{L}i\end{array})$$

Similarly, the probability to be heterozygous for the allele “*A*” (*ρ*_*Aa*_) for any individual of class *i* is:

9 $${\rho }_{Aa,i}^{{n}_{L}}=(\begin{array}{c}2{n}_{L}-2\\ 2{n}_{L}i-1\end{array})/(\begin{array}{c}2{n}_{L}\\ 2{n}_{L}i\end{array})$$

Finally, the probability to be homozygous for the allele “*a*” (*ρ*_*aa*_) for any individual of class *i* is computed as:

10 $${\rho }_{aa,i}^{{n}_{L}}=(\begin{array}{c}2{n}_{L}-2\\ 2{n}_{L}i-2\end{array})/(\begin{array}{c}2{n}_{L}\\ 2{n}_{L}i\end{array})=1-{\rho }_{Aa}^{{n}_{L}}-{\rho }_{AA}^{{n}_{L}}$$

Equations (8), (9) and (10) can be used to add a selective advantage in a second gene or to recognize a specific genotype in the range of possible genetic classes. The probability of a class *i* to carry a specific genotype is computed as:

11 $${\rho }_{genotype,i}=\prod _{m=1}^{{n}_{L}}{\rho }_{allele,\tfrac{2{n}_{L}i-{\Delta }_{allele}}{2({n}_{L}+1-m)}}^{{n}_{L}+1-m}$$

Where *ρ*_*allele*_ denotes the probability to be homozygous (*ρ*_*AA*_ or *ρ*_*aa*_) or heterozygous (*ρ*_*Aa*_) for upper cases or lower case alleles at the position of the gene *m*. The parameter Δ_*allele*_ represents the subtraction of lower case alleles at the previous genes. It subtracts 2, 1 or 0 alleles depending on the genetic characteristics of the previous gene *m *− 1, i.e. *aa*, *Aa*, *AA*, respectively. For instance, the probability to have a genotype “*AABbCcddEe*” in the phenotype class 0.5 (on a target genome with five genes), would be computed as: $${\rho }_{AABbCcddEe,0.5}=$$$${{\rho }^{5}}_{AA,\tfrac{5}{10}}{{\rho }^{4}}_{Aa,\tfrac{5}{8}}{{\rho }^{3}}_{Aa,\tfrac{4}{6}}{{\rho }^{2}}_{aa,\tfrac{3}{4}}{{\rho }^{1}}_{Aa,\tfrac{1}{2}}$$.

The final number of offspring of type *i* is then calculated as the addition of all breeding events leading to the same type of genotype class *i*, as follows:

12 $${n}_{k(t)}=\sum _{i,j\in [0,\mathrm{...},1]}{B}_{ij,k(t)}$$

To evaluate the temporal dynamics of adult populations after intra and interspecific density-dependent competition, we used a logistic regulation. We take into account the “lattice effect”^40^, in which the final outcome could be perturbed by the non-continuous nature of the number of individuals in a given population. This is incorporated through the rounding off of the following recursion equation:

13 $${N}_{i(t+1)}=round\,[{n}_{i(t)}[1+{r}_{i}({K}_{i}-\sum _{j\in [0,\ldots ,1]}{\alpha }_{ij}{n}_{j})/{K}_{i}]]$$

Where ${K}_{i}$ represents the carrying capacity and ${r}_{i}$ the growth rate *per capita* of the *i*^th^ genetic class. The value of *α* represents the intra- (*α*_*ii*_) and inter-class competition effects (*α*_*ij*_). This value is usually set to 1 for intraspecific competition of parental organisms (*α*_00_ or *α*_11_). Therefore, zero represents no competition for environmental resources, while a full competition is achieved with a value of 1. Those values may either be fixed for each class, or may depend on local population densities^12^, or being conditional to the genomic overlap from parental species. In this last case, the values of *α*_*ij*_ may be computed the same way as parameter *γ*_*ij*_ in equation 2.

In the simulations that we present in this paper we used interbreeding (γ) and competition (*α*) depending on the proportion of loci coming from the parental classes 0 and 1. In the case of competition, parental organisms may or may not be in competition for environmental resources (*α* ≠ 0 or *α* = 0, respectively), but full competition will always be acting on individuals of the same genotype, and for hybrids of different genetic classes, which level will depend on the proportion of shared loci.

### Model exploration

Because various previously developed models focused on one single locus^24–26^, we first assessed the influence of the number of simulated genes on the time needed to reach equilibrium, defined as the moment when frequencies of all classes are stable between two consecutive generations. We also estimated the deviation from the Hardy-Weinberg (HW) equilibrium at the time when simulations reach frequencies at equilibrium.

We explored the expression of particular loci (i.e. qualitative expression) and additive genes (i.e. quantitative expression), emerging when two genetically distinct individuals hybridize. We first focused in a virtual quantitative trait influenced by additive neutral genes. In this case, the genotypic classes, with different proportions of genes from parental species, can be considered as phenotypes expressed from the additive genes. We used our model to assess the range of possible phenotypes susceptible to be observed (i.e. the phenotypic landscape). We then introduced a deleterious allele for which we set a fitness reduction of 30%. Deleterious alleles with such a level of fitness loss are already being documented^41^. We further explored the effect of fitness reduction in the whole range of possible values and its interaction with the demographic effect.

We also examined the influence of different population sizes (i.e. carrying capacities) and competitive abilities between parental species on the phenotypic landscape and evaluate the risk of species extinction by genetic swamping that can be reached with the combined effects of genetic and demographic factors.

### Hybridization with mallards

The demographic parameter values are based on a previous publication characterizing the population dynamic of mallards (Table 1)^42^. We use those parameters to explore the situation in which mallards are seriously threatening local species by introgression. One of the most emblematic cases is the grey duck (*A. superciliosa*) in New Zealand, which is seriously threatened by mallards that were introduced for hunting at the end of the 19^th^ and early 20^th^ century^43^. The decrease of this native species and the appearance of hybrid phenotypes are well documented. Several populations of grey duck are already extinct and most current individuals have a hybrid ancestry^44^. In the 80’s, Gillespie^33^ estimated the proportion of the total population with pure native genome to be 4.5%, the proportion of grey duck-like hybrids at 11.7%, and mallard-like hybrids at more than 60%. He recognized seven phenotypic categories among hybrids and pure parental individuals, for which the relative frequencies are documented. Because we have *2n*_*L*_ + 1 phenotypic classes in our approach, to examine this case, we adapted the parameters of our model by assuming that the phenotypic differentiation is the product of three additive genes (*n*_*L*_ = 3). We also considered a similar population growth rate in both taxa, and we tried to estimate other demographic and genetic parameter values that best explain the invasion of mallards and the decline of the native species. We simulated the genetic interaction to begin in 1910, when the feral population of mallards was established^43^. In order to explain the observed hybrid frequencies, we set full interspecific competition of mallards (α_mallard_ = 1) to assess the carrying capacity (*K*) and the amount of interspecific competition performed by the native species (α_native_). We focused on the parameters of competition and carrying capacity because the competitive exclusion of native taxa by mallards due to their aggressive behaviour and the use of generalist habitats has been documented^31^. We thus explored different values of interbreeding success rate (γ), competition (*α*) and carrying capacity (*K*) to find the combination of values that best explain the history of colonization and hybridization of mallards in New Zealand, using the abundances reported by Gillespie^33^ as references. The best-explained scenario was selected as one with the minimal mean absolute error between observed and predicted values, computed as: $\sum |{x}_{predicted}-{x}_{observed}|/n$. We then used this best-explained scenario to project 50 additional generations of interbreeding in order to investigate future consequences. All simulations were performed using *R*^45^.

**Table 1:** List of main functions and parameters used in our general hybridization model.

Notation	Definition
***List of functions***	
*N*_*i*_	Number of adult individuals of phenotypic class *i*
*M*_*ij*_	Mating probability between phenotypic classes *i* and *j*
*b*_*ij,k*_	Number of mating *i x j* resulting in offspring of type k
*C*_*ij,k*_	Probability of mating *i x* j to produce offspring of types *k*
*ω*_*i*_	Effect of inherited characters on fitness of individuals of class *i*
*ρ*_*Aa,i*_	Probability of class *i* to be heterozygous for the allele “*A”*
*n*_*k*_	Total number of offspring of class *k*
***Demographic parameters*** ^*^	
*K*	Carrying capacity
*r*	Growth rate *per capita*
*α*	Competition
***Intercross parameters***	
*n*_*L*_	Number of independent biallelic loci
*γ*	Interspecific success rate
*Ω*_*A*_	Relative fitness of allele “*A*” in relation to the alternative form “*a*”
*ε*_*A*_	Dominance degree of the allele “*A*”

^*^Demographic parameter values of carrying capacity and growth rate were fixed according to previous simulations based on a 40-years survey of mallards (*K = *118.8, *r = *1.3) (see^42^).

### Data accessibility statement

The R code for this study is available at: http://genev.unige.ch/montoya-currat/scripts/.

## Electronic supplementary material

Supporting information
