# Supplementary material for: Cryptic Biological Invasions: a General Model of Hybridization
Source: Sci Rep. 2018 Feb 5;8:2414. doi: 10.1038/s41598-018-20543-6 (PMC5799175; doi:10.1038/s41598-018-20543-6)
Supplement: Supplementary file 1 — Supporting information [file 41598_2018_20543_MOESM1_ESM.pdf]

Scientific Reports

Supporting information

Cryptic Biological Invasions: a General Model of Hybridization

Claudio S. Quilodrán, Frédéric Austerlitz, Mathias Currat, Juan I. Montoya-Burgos

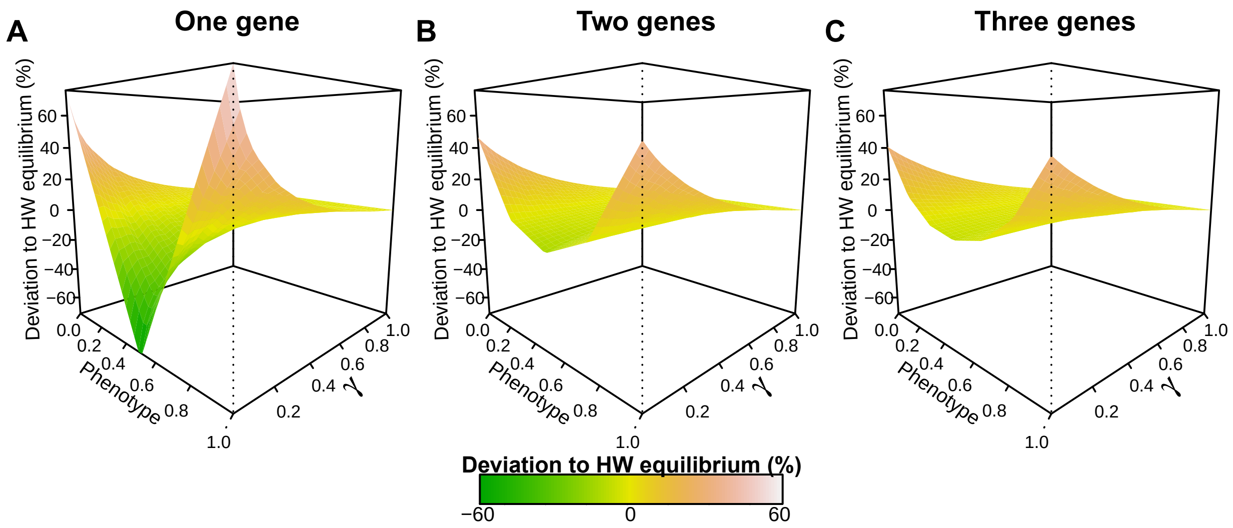

Figure S1. The effect of different number of simulated genes on the deviation to the Hardy-Weinberg (HW) equilibrium reached at the time of frequencies in equilibrium. We explore values of interbreeding success rate ranging from rare hybridization success ($\gamma = 0.01$) to a panmictic mating system ($\gamma = 1$). The data are exposed after 150 generations of genomic mixing.

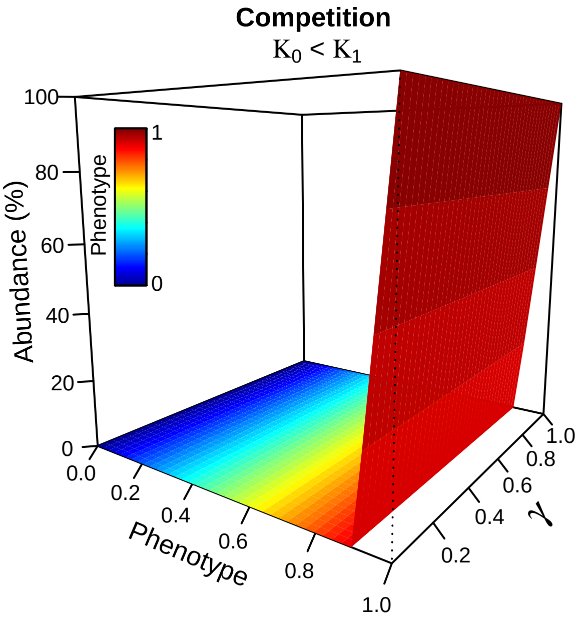

Figure S2. Density-dependent demographic parameters influencing the landscape of observed phenotypes. The phenotypic landscape is extrapolated from the contribution five additive genes ($n_L = 5$). We show the effect of interspecific competition ($\alpha_{01} = \alpha_{10} \neq 0$) with unequal carrying capacities between the two parental species ($K_1 = 1.5K_0$). We explored various levels of interbreeding success rate, from rare hybridization success ($\gamma = 0.01$) to a panmictic mating system ($\gamma = 1$). The data are presented after 100 generations of independent evolution and 150 generations of hybridization (250 total generations)

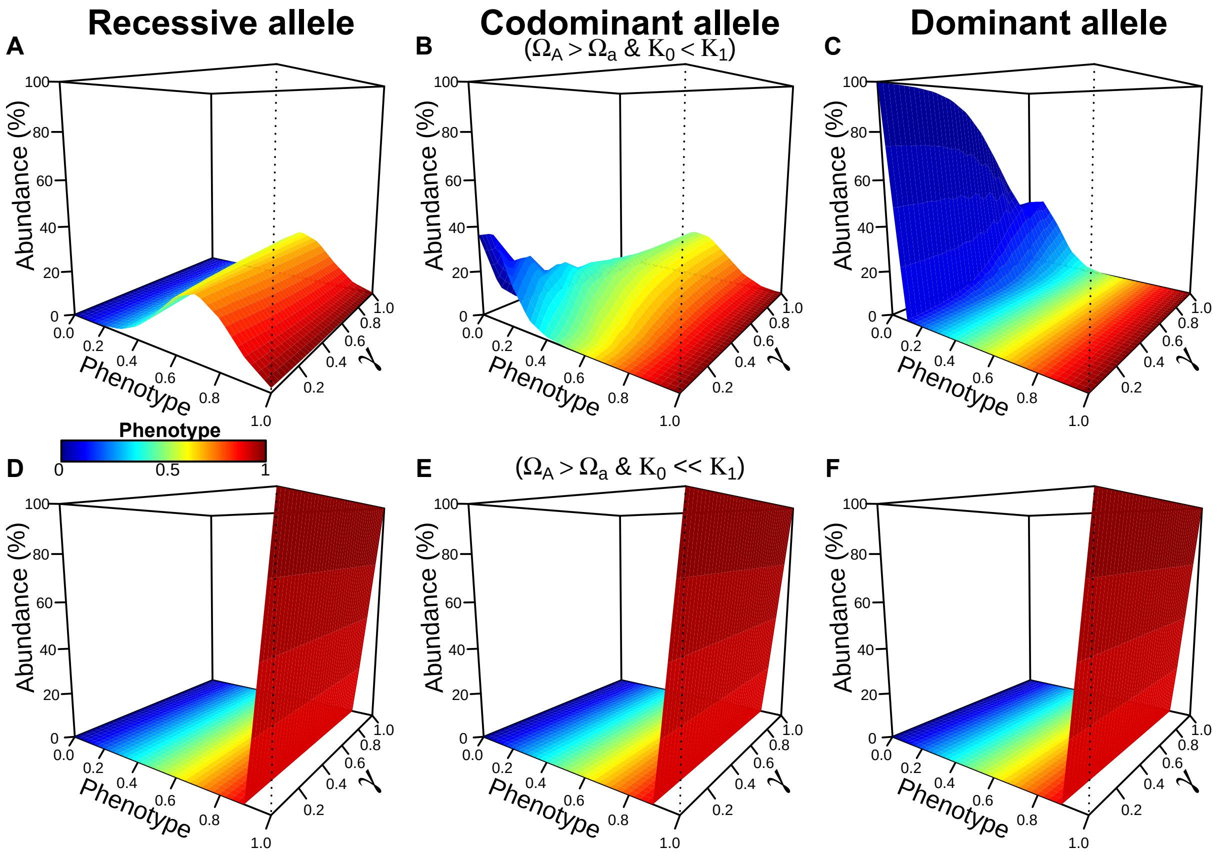

Figure S3. Effects of a deleterious allele "a" with fitness reduced by 30%. There is interspecific competition between parental taxa ($\alpha_{01} = \alpha_{10} \neq 0$). The parental species with reduced allelic fitness (species of class 1) has higher or much higher carrying capacities than the other parental species (class 0) ($K_1 = 1.5K_0$ and $K_1 = 2K_0$). The results are obtained after 100 generations of independent evolution and 150 generations of genomic mixing (250 total generations).

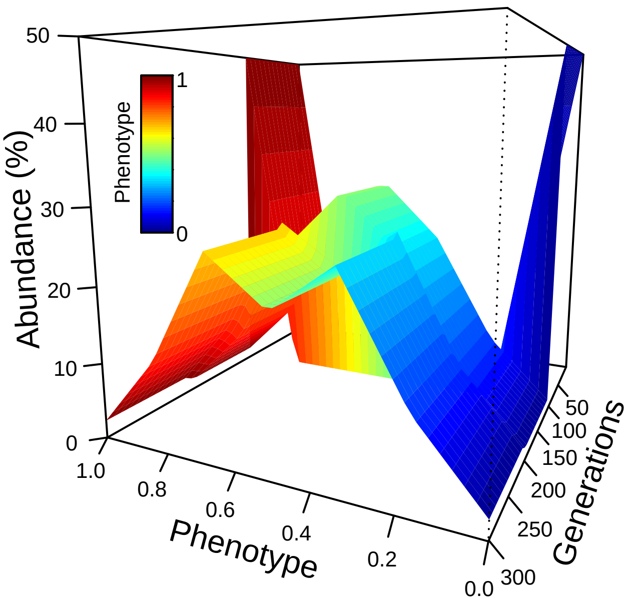

Figure S4. Forward simulations of the abundance of two parental species (classes 0 and 1), which have experienced independent evolution during 100 generations before hybridization starts. The interbreeding is panmictic ($\gamma = 1$). A lethal genotype ("*AaBbCc*") appears in generation 200 ($n_L = 3$).

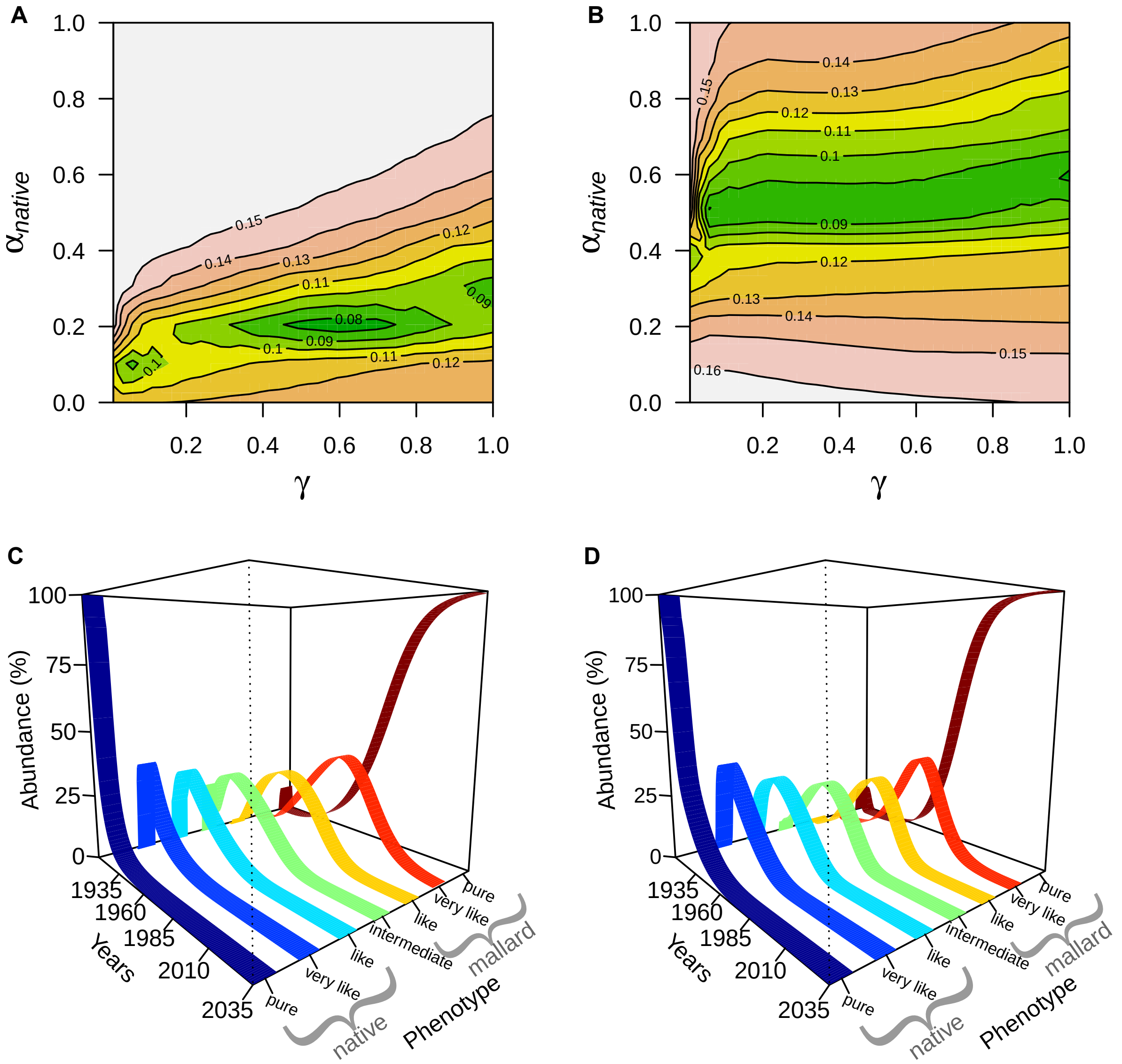

Figure S5. Parameter values and forward simulations when using alternative combinations of carrying capacities to explain the observed colonization of mallards (*Anas platyrhynchos*) in New Zealand. a) and c) carrying capacity of mallard 1.75 times larger than the one of the native species; b) and d) carrying capacity of mallard 1.5 times larger than the one of the native species.
